# Does *Cedrela* always form annual rings? Testing ring periodicity across South America using radiocarbon dating

**DOI:** 10.1007/s00468-017-1604-9

**Published:** 2017-08-20

**Authors:** Jessica C. A. Baker, Guaciara M. Santos, Manuel Gloor, Roel J. W. Brienen

**Affiliations:** 1 School of Geography, University of Leeds, Leeds, UK; 2 Earth System Science, University of California, Irvine, USA

**Keywords:** Tree rings, Growth rhythms, ^14^C, Amazon, Biannual ring formation

## Abstract

**Key Message:**

**Radiocarbon dating shows that** ***Cedrela*** **trees from Bolivia, Ecuador and Venezuela form one ring per year but** ***Cedrela*** **trees from Suriname form two rings per year.**

**Abstract:**

Tropical tree rings have the potential to yield valuable ecological and climate information, on the condition that rings are annual and accurately dated. It is important to understand the factors controlling ring formation, since regional variation in these factors could cause trees in different regions to form tree rings at different times. Here, we use ‘bomb-peak’ radiocarbon (^14^C) dating to test the periodicity of ring formation in *Cedrela* trees from four sites across tropical South America. We show that trees from Bolivia, Ecuador and Venezuela have reliably annual tree rings, while trees from Suriname regularly form two rings per year. This proves that while tree rings of a particular species may be demonstrably annual at one site, this does not imply that rings are formed annually in other locations. We explore possible drivers of variation in ring periodicity and find that *Cedrela* growth rhythms are most likely caused by precipitation seasonality, with a possible degree of genetic control. Therefore, tree-ring studies undertaken at new locations in the tropics require independent validation of the annual nature of tree rings, irrespective of how the studied species behaves in other locations.

## Introduction

Tropical dendrochronology is a steadily growing field, and the number of species known to be suitable for tree-ring analysis is also rising. Annual ring formation has now been shown in 230 tropical tree species (Brienen et al. 2016b), providing a great opportunity to further develop tropical tree-ring research. Tropical tree rings and their associated characteristics can be used to reconstruct climate (e.g. Baker et al. 2016; Mendivelso et al. 2013; Schöngart et al. 2006; Vlam et al. 2014; Xu et al. 2015), inform sustainable forest management (e.g. Brienen and Zuidema 2006; De Ridder et al. 2013; Schöngart 2008), study forest dynamics (e.g. Brienen et al. 2010; Vlam et al. 2017) and potentially to monitor forest responses to climate change (e.g. van der Sleen et al. 2015; Zuidema et al. 2012, but also see Brienen et al. 2016a). With such important applications, it is vital to understand what drives ring formation and thus how growth dynamics might vary between sites, even within a single species.

Temperature, which induces cambial dormancy and ring formation at high latitudes, has limited seasonality in the tropics (Jacoby 1989), and seasonal variation in rainfall is instead thought to be the most common cue for growth periodicity and tree-ring formation (Brienen et al. 2016b). During an extreme dry period, water stress can result in cambial dormancy and this may be accompanied by discernible changes to the structure of the xylem, thus resulting in a growth band or tree ring (Bräuning et al. 2008b; Dünisch et al. 2002; Mendivelso et al. 2013; Worbes 1999, 2002). Deciduous trees shed their leaves in response to the water stress, only to flush their leaves again and thereby reactivate the cambium once tree water status has been restored (Borchert 1999). Periodic flooding and the ensuing anoxia can provide a similar trigger for ring formation in floodplain tree species (Schöngart et al. 2002). However, several studies have also highlighted the important influence of seasonality in daily insolation (amount of solar radiation per unit area) and photoperiod on tropical tree phenology, with phenological changes (such as shedding/flushing leaves) sometimes occurring in advance of the climate changes (such as water stress/onset of rains) that the trees might be expected to respond to (Borchert et al. 2005, 2015; Elliott et al. 2006; Lisi et al. 2008; Rivera et al. 2002). Furthermore, besides external cues, intrinsic plant rhythms are likely to play some role in governing cambial activity (e.g. Callado et al. 2013; Villalba 1985). This shows that identifying the trigger factor for growth rhythms in tropical trees is not always straightforward, as different tropical tree species respond to different cues (Borchert et al. 2015), and there could also be differences in response between sub-populations of the same species (e.g. Ruiz et al. 2013; Stubblebine et al. 1978).

Although the exact environmental trigger for ring formation in tropical trees may still be under discussion, it follows that regional variation in the stimulus may cause variation in growth periodicity. Indeed, previous work in the tropics has shown that a single species may have different growth rhythms under different environmental regimes (Costa et al. 2013). A species that forms distinct annual rings at a location with seasonal precipitation may form vague or false (non-annual) rings at a location with low or irregular precipitation seasonality, or may not form visible growth rings at all (Borchert 1999; Boysen et al. 2014; Pearson et al. 2011; Priya and Bhat 1999). Tree-ring formation may, therefore, occur at regular intervals (i.e. annual/biannual rings) or at irregular intervals (intermittent false rings) depending on the seasonality of environmental conditions (Gourlay 1995; Jacoby 1989). Thus, care should be taken when analysing tree rings from a new species, or from a known species in a new location (Brienen et al. 2016b).

Tree-ring periodicity can be tested using ‘bomb-peak’ radiocarbon (^14^C) dating. Thermonuclear tests during the late 1950s caused an artificial increase in atmospheric ^14^C (peaking around 1963–1964), which has slowly been removed from the atmosphere since the 1962 Test Ban Treaty (Levin et al. 2008). From 1950 onwards, atmospheric ^14^C signatures have been recorded across the globe at sites away from localized emissions sources, such as large cities and volcanoes, and despite small variations (e.g. at the onset of the thermonuclear tests), these signatures are mostly well distributed across the hemispheres (Hua et al. 2013; Levin et al. 2008; Levin and Hesshaimer 2000). This means that organic material from the last 60 years can be dated with an accuracy of 1–2 years by measuring its ^14^C content, providing a means to validate tree-ring dates (e.g. Andreu-Hayles et al. 2015; Bormann and Berlyn 1982; Pearson et al. 2011; Santos et al. 2015; Worbes and Junk 1989).

This study focuses on *Cedrela odorata* and its highland relative *Cedrela montana*. *Cedrela* spp. have been used extensively in tree-ring studies in South America and are widely believed to form annual rings (e.g. Ballantyne et al. 2011; Bräuning et al. 2009; Brienen and Zuidema 2005; Costa et al. 2013; Dünisch et al. 2002; Espinoza et al. 2014; Tomazello-Filho et al. 2000; Worbes 1999). Our aim is to use bomb-peak ^14^C dating to test the annual character of tree rings from four sites across the Amazon basin that vary in their precipitation and insolation seasonality. We complement this analysis with tree-ring and growth rhythm data of *Cedrela* from various additional sites from Central and South America, and discuss what might be driving the observed variability in tree-ring periodicity.

## Methods

**Fig. 1:**
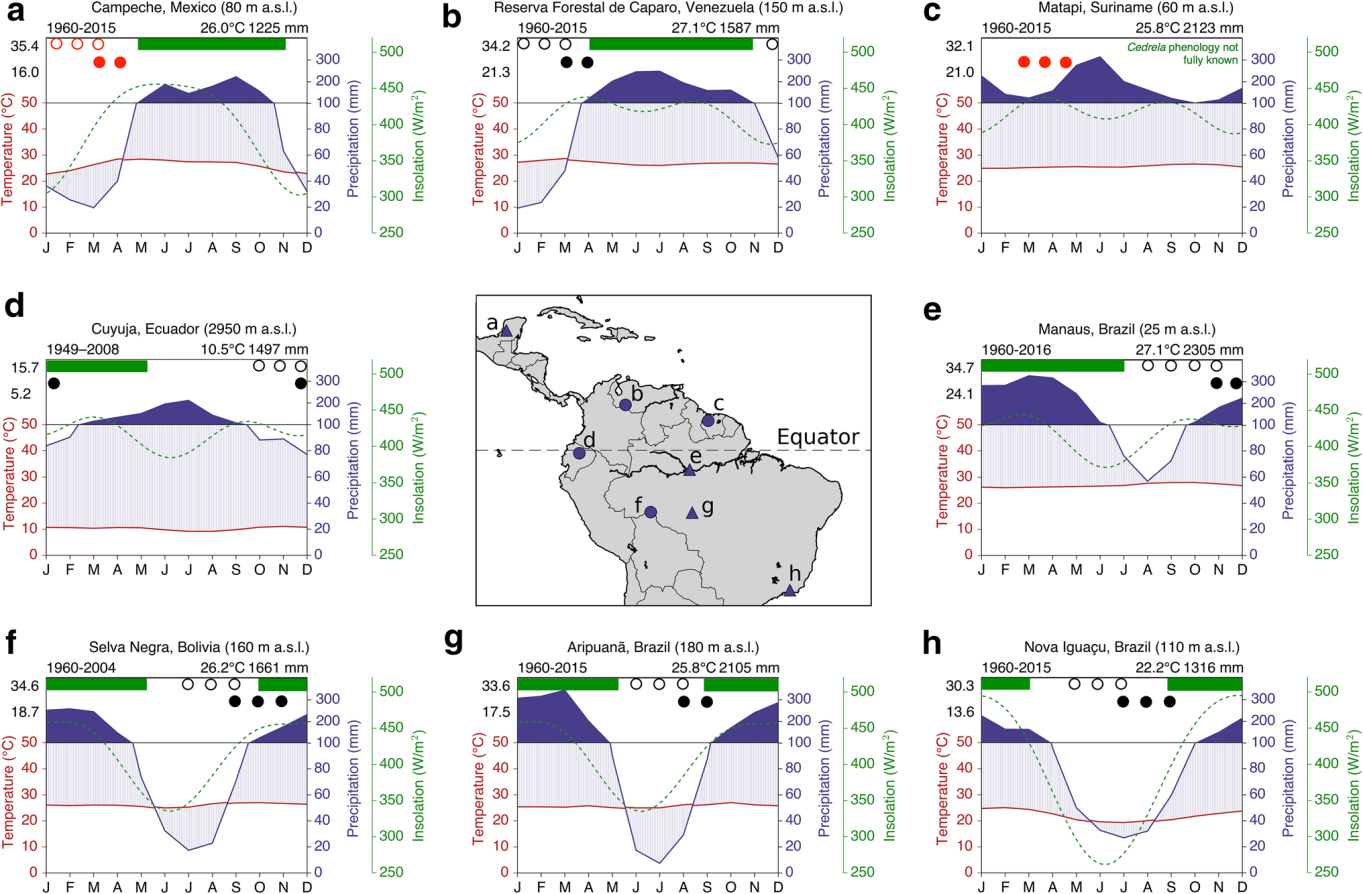
Map showing the locations and climate diagrams of the four sites sampled in this study (*dark blue circles*) and four sites where *Cedrela* growth periodicity data are available from the literature (*dark blue triangles*). The altitude of each site is given in m above sea level (m a.s.l.). The date range at the *top left* of each climate diagram is the period of temperature and precipitation observations. The values in *black* at the *top right* are the mean annual temperature and the total annual precipitation. Values in *black* on the *left side* of each diagram are the maximum and minimum annual temperatures. Note that the precipitation scale changes above 100 mm, indicated by a change to *solid blue fill*. Average daily insolation data are also shown (*dashed green line*). *Horizontal green bars* at the *top* of each graph show the main growing period for *Cedrela* at each of the sites. *Open* and *filled black circles* show the periods of *Cedrela* leaf-fall and leaf-flush, respectively, with data from systematic monitoring. *Open* and *filled red circles* show the periods of *Cedrela* leaf-fall and leaf-flush, respectively, with data from casual observations. Sources for the climate, insolation, growth and phenology data used for each site are given in the “Methods”

The samples used in this study came from four locations across the Amazon basin: Reserva Forestal de Caparo in Venezuela [7.45°N, 70.98°W, 150 m above sea level (a.s.l.)], a logging concession near Matapi, Suriname (4.90°N, 56.85°W; 60 m a.s.l.), Cuyuja, Ecuador (0.45°S, 78.04°W; 2950 m a.s.l.), and Selva Negra, Bolivia (10.10°S, 66.31°W; 160 m a.s.l.). These locations and their corresponding climate diagrams are shown in Fig. 1. Four additional sites where *Cedrela* growth data are available from the literature are also shown for comparison: Manaus, Amazonas State, Brazil (Dünisch and Morais 2002), Aripuanã, Mato Grosso State, Brazil (Dünisch et al. 2003), Nova Iguaçu, Rio de Janeiro State, Brazil (Costa et al. 2013) and Ejido Pich, Campeche State, Mexico (Brienen et al. 2010). Temperature and precipitation data are from local weather stations or extracted from the Climatic Research Unit (CRU) TS3.24 0.5° × 0.5° dataset (Harris et al. 2014) and downloaded via Climate Explorer (Trouet and Van Oldenborgh 2013). Daily insolation data were downloaded from the National Aeronautics and Space Administration website (http://data.giss.nasa.gov/ar5/srlocat.html) and averaged over the period 1990–2000. Growth and phenology data are also shown in Fig. 1. Sources of these data are as follows: Mexico (Brienen et al. 2010), Venezuela (Worbes 1999), Suriname (personal communication, P. Teunissen), Ecuador (Bräuning et al. 2009), Bolivia (Brienen and Zuidema 2005) and Brazil (e.g. Manaus (Dünisch and Morais 2002), Aripuanã (Dünisch et al. 2003) and Nova Iguaçu (Costa et al. 2013)).

There are some subtle differences in climate between the sites in this study. While temperature shows little seasonality at any of the sites, there is some variability in rainfall regime. Mexico, Venezuela, Bolivia, Aripuanã and Nova Iguaçu all have one pronounced dry season, with precipitation falling below 50 mm for three consecutive months or more (Fig. 1a, b, f–h). Ecuador and Manaus also have a single distinct dry season when monthly precipitation falls below 100 mm for at least three consecutive months (Fig. 1d, e). In Suriname, precipitation has a bimodal distribution, peaking in January and June, and does not fall below 100 mm in any month (Fig. 1c). Insolation distributions also vary, with sites furthest from the Equator experiencing a single annual insolation peak (i.e. Fig. 1a, f–h) and sites close to the Equator experiencing two insolation peaks in each year (i.e. Fig. 1b–e).

*C. odorata* was sampled in Bolivia, Venezuela and Suriname, and the closely related species *C. montana* was sampled in the high-elevation site in Ecuador (hereafter referred to by genus name only). Stem discs were collected in 2011 (Bolivia), 2013 (Ecuador) and 2014 (Suriname) from trees felled for timber or during the installation of overhead power lines. The Venezuelan samples were collected in 2012, using an increment borer to collect cores from living trees. Discs were polished using an orbital sander with sandpaper up to grit 600 to improve ring visibility. On each disc rings were marked on 2–4 radii and every 10th ring was interconnected between radii to crosscheck counting accuracy, and account for wedging rings (Brienen et al. 2016b). A core-microtome (Gärtner and Nievergelt 2010) was used to prepare the surface of the cores from Venezuela. Rings on the cores were then marked, measured using a LINTAB measuring stage to the nearest 0.01 mm, and visually crossdated across 2–3 radii. Disc sections (cut using a bandsaw) and cores were then scanned at high resolution using an Epson Expression 11000XL scanner (Fig. 3a–c). As the rings were particularly narrow on the samples from Suriname, the microtome was used to cut thin sections (~10 µm thick) that were scanned with an Epson Perfection V700 Photo scanner (Fig. 3d) to optimise ring visibility. Rings on the samples from Suriname were observed to frequently follow a regular pattern of a narrow ring followed by a wide ring, possibly indicating the presence of non-annual rings (e.g. Gourlay 1995). Where this pattern was identified, the narrow rings were assumed to be false and thus the wide and narrow rings were initially counted together as a single annual ring and dated accordingly. All rings were dated following the convention of Schulman (1956), where the assigned calendar date corresponds with the year that the tree started growing. Finally, although radii within each tree were crossdated, a conventional inter-tree crossdating analysis was not conducted for this study. This was because previous work has shown that trees from Bolivia show only a weak correspondence of ring-width patterns between trees (e.g. *r*_mean_ = 0.17, EPS = 0.64; Baker et al. 2015), even when rings showed very strong oxygen isotope (δ^18^O) crossdating statistics (*r*_mean_ = 0.71, EPS = 0.96; Baker et al. 2015) and strong climate-δ^18^O relationships (see Baker et al. 2016). Trees from the other sites had a high incidence of strongly wedging rings, which weaken inter-radial ring-width relationships and, thus, between-tree correlations were also expected to be weak.

To independently validate this initial ring age-assignment, between one and three trees from each site were selected for bomb-peak ^14^C dating. This approach is a useful method for validating crossdated tree-ring samples (e.g. Andreu-Hayles et al. 2015; Baker et al. 2015; Bormann and Berlyn 1982; Pearson et al. 2011; Santos et al. 2015; Worbes and Junk 1989). All measurements were performed on α-cellulose or holocellulose extracts rather than whole wood, as these wood fractions are immobile and will thus produce more precise radiocarbon dates than if whole wood were used (Leavitt and Bannister 2009). For each sample, 2–4 rings putatively dated from 1955 to 1985 were selected for analysis, and the wood cut from each individual ring using a scalpel (in total 25 samples from 8 different trees). Cellulose extraction for the Suriname samples was conducted in Leeds, following the batch method of Wieloch et al. (2011). These samples were then sent for ^14^C analysis by means of accelerator mass spectrometry (AMS) in Bothell, USA by DirectAMS (http://www.directams.com). AMS analysis used NIST Ox-II standards (Stuiver 1983) for normalization, and IAEA-C7 as secondary standards (Le Clercq et al. 1997). Graphitisation of CO_2_ produced by combustion of organic materials was via the zinc reduction method (Vogel 1992). All other samples were analysed at the W. M. Keck Carbon Cycle Accelerator Mass Spectrometer (KCCAMS Facility) located at the Earth System Science Department at the University of California in Irvine, USA. At KCCAMS holocellulose was isolated following a method adapted from Leavitt and Danzer (1993) with AnkomTM F57 Dacron filter bags (25 µm effective pore size) used as sample pouches. Wood samples loaded in pouches were lined up in a Soxhlet apparatus and initially treated with a 2:1 mixture of >99.5% toluene and high-performance liquid chromatography (HPLC) grade ethanol for 24 h, and later by pure HPLC ethanol for another 24 h. Subsequent processing used hot Milli-Q water to remove solvent residues, followed by bleaching at 70 °C with a sodium chlorite solution acidified with 2 ml of 100% glacial acetic acid. Once samples turned white, they were washed with Milli-Q water and gently dried at 50 °C in a conventional drying oven. After extraction of holocellulose, samples were removed from pouches, combusted and graphitized following established protocols (Santos et al. 2007). Wood blank (^14^C-free) and secondary standards (FIRI-J and FIRI-H; Scott 2003), as well as cellulose extract (IAEA-C3; Rozanski et al. 1992), were processed alongside samples for background corrections and quality control purposes. High-precision ^14^C measurements were conducted at an in-house modified AMS compact instrument (Beverly et al. 2010) via multiple analyses of the primary and normalizing standard Oxalic Acid I (OX-I; Olsson 1970). All ^14^C results were corrected for background effects and isotopic fractionation due to photosynthesis, sample processing and spectrometer analysis, with δ^13^C of prepared graphite measured directly at the spectrometer, as described by Santos et al. (2007).

To check the accuracy of the individual tree-ring dates, their respective fraction modern carbon values (F^14^C, defined as the ratio of the radioactivity of the sample to the radioactivity of the modern standard; Reimer et al. 2004) were plotted alongside atmospheric radiocarbon bomb-peak calibration curves from designated zones in the Northern and Southern Hemispheres (e.g. NHZ2 and SHZ3; Hua et al. 2013). The calendar dates that had been assigned initially (following Schulman’s convention, see above) were converted to a decimal date that was centred in the middle of the growing season for each site (green bars in Fig. 1), as this is when trees photosynthesise atmospheric ^14^CO_2_ and form tree-ring cellulose. For example, the ring 2000 would be adjusted to 2001.0, 2001.25 and 2000.5 in samples from Bolivia, Ecuador, and Venezuela, respectively, as these dates fall within the growing season at each location (see growth data sources above). Samples from Suriname were not adjusted relative to the initial assigned dates, as the main growing period for *Cedrela* is unknown for this site.

**Fig. 2:**
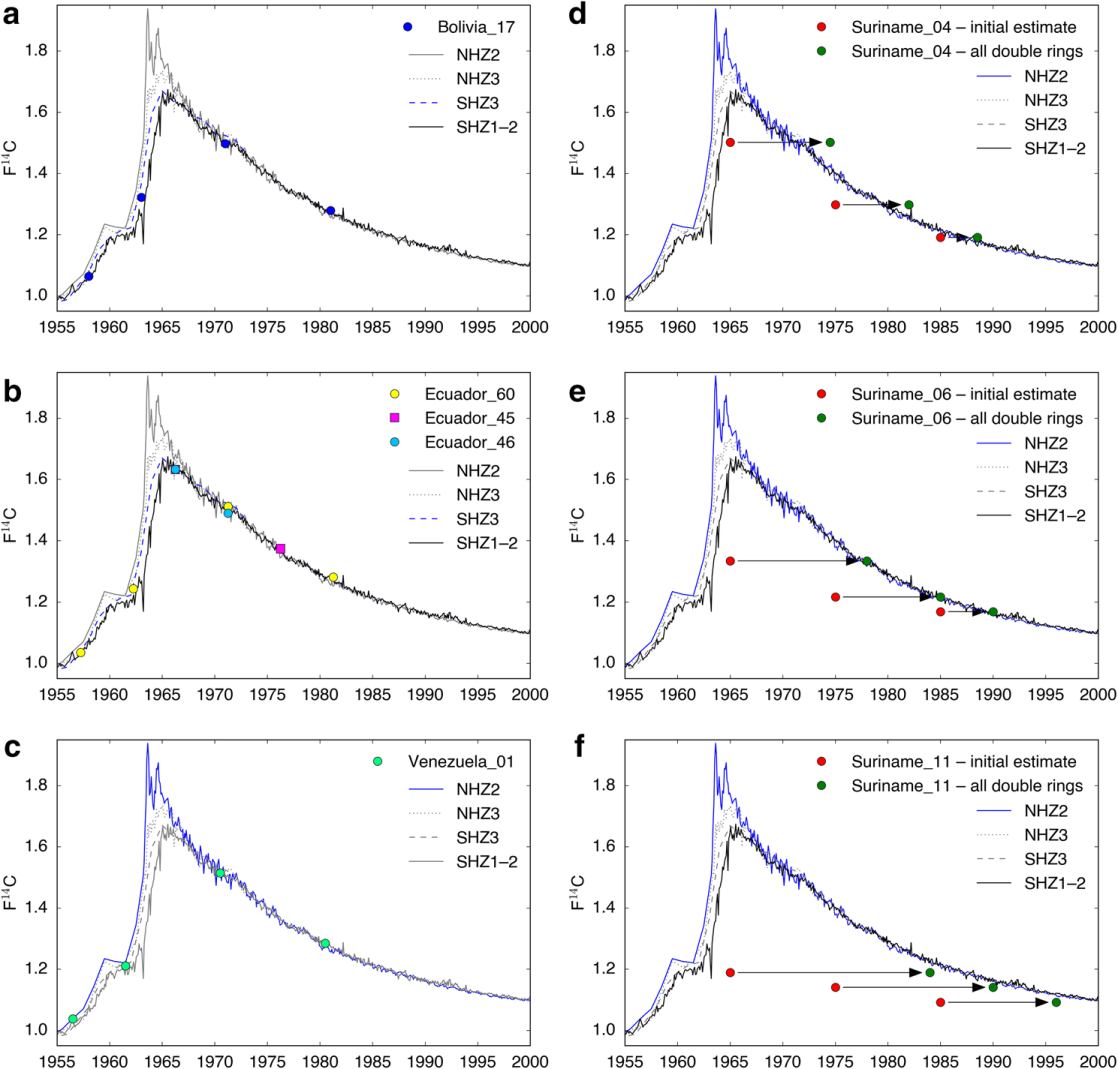
F^14^C values from ^14^C-AMS analysis (*circles*) plotted over the northern hemisphere (NH) and southern hemisphere (SH) ^14^C bomb datasets compiled by Hua et al. (2013). **a**–**c** Samples from Bolivia (tree 17), Ecuador (trees 45, 46 and 60) and Venezuela (tree 01) all fall on top or between these calibration curves. **d**–**f** Samples from Suriname (trees 04, 06 and 11) plotted using the initial sample age estimates (*red circles*) and the age estimates if trees are assumed to regularly form two rings per year (*green circles*). In each panel, the location-specific atmospheric ^14^C calibration curve presently accepted by the radiocarbon community (as per Hua et al. 2013) is highlighted in *blue*

**Fig. 3:**
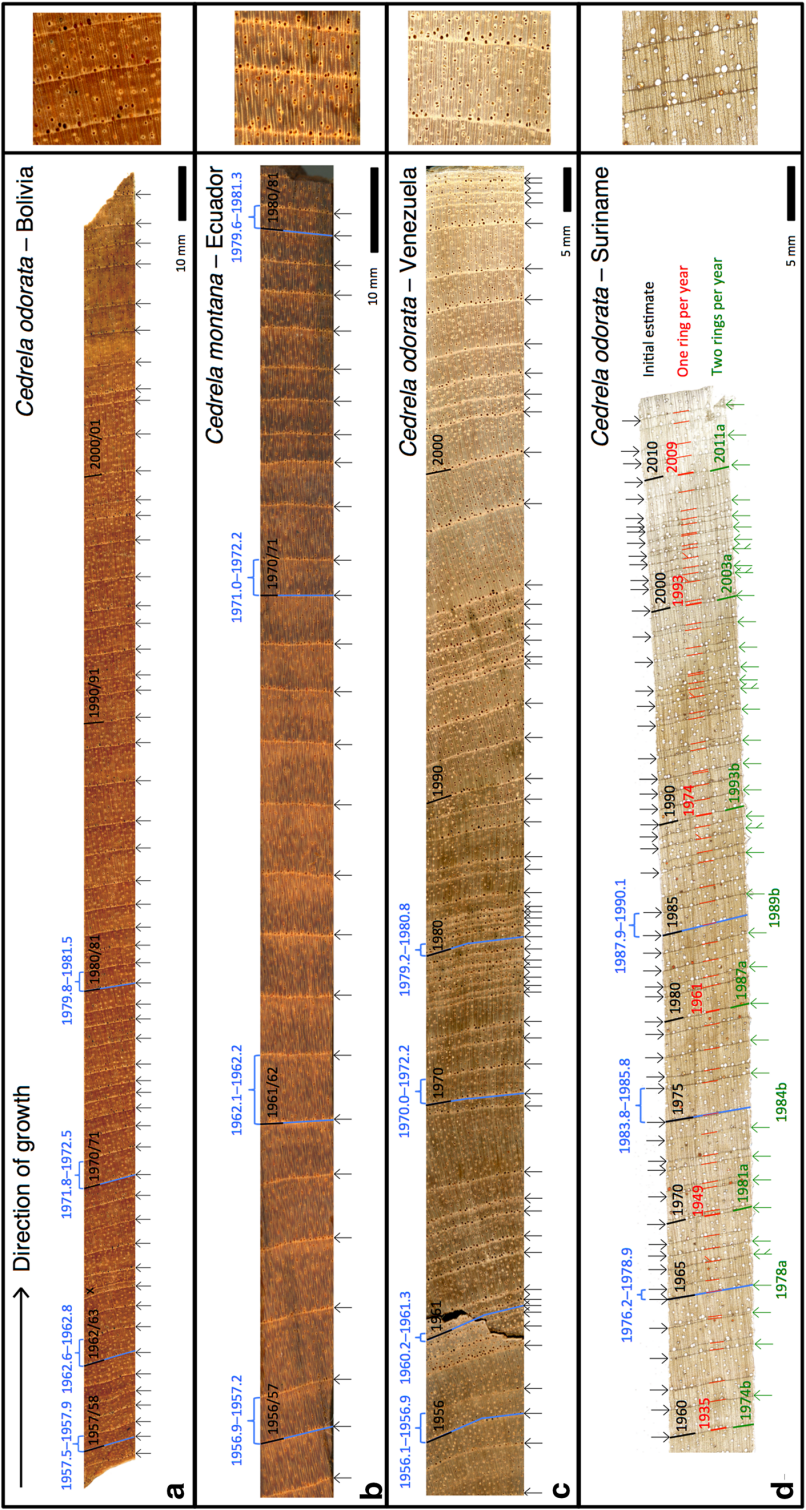
High-resolution scans of one sample from **a** Bolivia (tree 17), **b** Ecuador (tree 60), **c** Venezuela (tee 01) and **d** Suriname (tree 06). *Black arrows* indicate the parenchyma bands counted as ring boundaries during the initial tree-ring dating process. For Suriname only, we show two extra annotations: ring boundaries if a tree forms one ring per year (*red markings*) or two rings per year (*green arrows*). *Blue brackets* and *lines* indicate the tree rings selected for ^14^C analysis with their initial assigned years shown in *black*. Note that a tree ring may grow across two calendar years (e.g. 2000/2001), depending on the main growing period at a particular location (see Fig. 1). Radiocarbon-derived calendar age ranges (±2σ) are shown in *blue* and were translated from the F^14^C values and uncertainties obtained from ^14^C-AMS using the free online CALIBomb software (http://calib.org/CALIBomb/) and the available intra-hemispheric datasets of Hua et al. (2013), e.g. SHZ3 (Bolivia and Ecuador) and NHZ2 (Venezuela and Suriname). New age estimates (assuming trees form two rings per year) are in *green*. When marking 2 rings per year the first and second rings are denoted *a* and *b,* respectively

Figures 1 and 2 were produced in Python 3.5.2 using the Scientific PYthon Development EnviRonment (Spyder) 3.1.2. Figure 3 was produced in Microsoft PowerPoint.

## Results and discussion

The measured F^14^C values were plotted alongside the atmospheric ^14^C calibration curves from Hua et al. (2013). The samples from Bolivia, Ecuador and Venezuela all fall on or between these curves (Fig. 2a–c), indicating that these trees have been accurately dated by counting rings and that *Cedrela* forms annual growth rings at each of these locations. High-resolution scans of samples from these locations illustrate the correspondence between tree-ring dates (black annotations) and radiocarbon dates (blue annotations, Fig. 3a–c). These results are consistent with previous tree-ring studies, which report annual ring formation in *Cedrela* spp. from Bolivia (Brienen and Zuidema 2005), Ecuador (Bräuning et al. 2009) and Venezuela (Worbes 1999). Furthermore, the excellent agreement between ^14^C in tree rings and existing bomb-peak calibration curves shows that well-dated tropical tree-ring records can potentially be used to refine low-latitude intra-hemispheric ^14^C calibration curves between 1950 and 1970, when distributions of atmospheric ^14^C were more variable across the globe. This could lead to present intra-hemispheric ^14^C calibration curves being redefined in tropical regions.

The Suriname samples were initially dated using a ‘best-guess’ approach, based on the assumption that a narrow ring followed by a wide ring should be counted as one single year. The initial estimated calendar dates fall far from the calibration curves (red circles, Fig. 2d–f), showing that the initial ring dating was inaccurate. The original samples were then re-inspected and the rings were dated assuming that the trees formed (i) a single ring each year and (ii) consistently two rings per year. Figure 3d shows a scan of Suriname sample 06 with the revised dates from these two approaches annotated in red (approach i) and green (approach ii). Only the second approach produced any sample dates close to the results from ^14^C analysis (blue annotations). When plotted against these adjusted dates (green circles), the F^14^C values fall either exactly on (Suriname_06, Fig. 2e) or closer to (Suriname_04 and Suriname_11, Fig. 2d, f) the bomb calibration curves. Samples from Suriname_04 appear to be overestimated by 1–3 years, while samples from Suriname_11 appear to be underestimated by 2–3 years. Nevertheless, these results are a strong indication that in Suriname *Cedrela* forms two rings every (or nearly every) year. The slight offsets that remain for Suriname_04 and Suriname_11 suggest that there could still be some minor dating errors with either one or two rings missed or miscounted. For example, in Fig. 2f the green circles fall to the left of the radiocarbon calibration curves, suggesting that there may have been one or two false rings in the Suriname_11 sample. To the authors’ knowledge, this is the first time that the formation of two rings per year has been reported in *Cedrela*, and shows that dendrochronologists should take a cautious approach when analysing samples from new sites in the tropics. Furthermore, if a species regularly forms two rings per year at a particular site, then conventional dendrochronological crossdating methods (see Stokes and Smiley 1968) may not detect that ring formation is not annual. Ring periodicity thus needs to be validated using radiocarbon dating, or by correlating a robust ring-width or isotope chronology against climate data over a sufficiently long period.

To explore what might be driving the observed spatial variation in growth periodicity, it is first necessary to understand how tree rings form in *Cedrela*. *Cedrela* is an obligate deciduous species and throughout most of its natural range (from Mexico to northern Argentina; Pennington et al. 1981) leaf shedding and associated dormancy occur strictly once per year, during the annual dry season (Fig. 1; Brienen and Zuidema 2005; Costa et al. 2013; Worbes 1999). Thus, in Bolivia, trees are leafless from July to September (Brienen and Zuidema 2005), in Ecuador from October to December (Bräuning et al. 2009) and in Venezuela from December to March (Worbes 1999), although the exact dates of leaf-fall and leaf-flush may vary between years. The cambium is inactive during this leafless period and a marginal parenchyma band marks the cessation of growth (Brienen and Zuidema 2005; Dünisch et al. 2002; Marcati et al. 2006). Ring structure in *Cedrela* is described as ring-porous or semi-ring-porous as large vessels form during reactivation of the cambium, resulting in an abundance of wide vessels embedded within the parenchyma band, and fewer, narrower vessels in the wood which forms later in the growing season (Dünisch et al. 2002; Vetter and Botosso 1989). This pattern is clear in samples from all of the sites in this study (side panels, Fig. 3). Leaf-fall behaviour of *Cedrela* in Suriname has not been systematically monitored so it is not known whether biannual ring formation corresponds to (or is induced by) biannual leaf exchange. Casual observations have been made of trees flowering and fruiting at the turn of the year (from September/October to February/March), and in 2010 and 2015, trees were observed to flush their leaves between March and May (personal communication, P. Teunissen). This is broadly similar to neighbouring Guyana where *Cedrela* flowers from August to November and fruiting occurs in January to March (Polak 1992; ter Steege and Persaud 1991), though leaf-fall behaviour has also not been consistently observed here. Despite this knowledge gap, we can still try to explore the factors controlling growth dynamics of tropical trees in an attempt to understand the regional variation in *Cedrela* ring formation reported here.

Growth periodicity in *Cedrela* may be driven by variation in an external environmental signal (e.g. precipitation or insolation), or by an intrinsic biological rhythm, which can each be considered for our sites. In Suriname, where *Cedrela* forms two rings each year, rainfall has a bimodal distribution. This is likely due to the oscillating position of the inter-tropical convergence zone (ITCZ) which follows maximum solar radiation and thus moves southward across the continent during austral summer and northward again during austral winter (Garreaud et al. 2009). For this reason, at the ‘climatic equator’, which in South America centres around 4–5°N, there may be two wet seasons as the ITCZ passes over two times in each year (Borchert et al. 2005). In contrast, the other seven sites show only one distinct wet and dry season per year, and at all seven of these sites *Cedrela* has been shown to form one ring each year. At these seven sites, the trees grow mainly during the wet season and are leafless during the driest part of the year (Fig. 1a, b, d–h). This provides a strong indication that tree rings at these sites are formed in response to seasonal water availability. The phenology of Suriname *Cedrela* has not been studied, so it is not known whether the species changes its leaves twice a year here, but it is possible that one of the two rings forms during a period of temporary cambial dormancy when the trees are still in leaf (Borchert 1999). However, it should also be noted that the seasonal water deficit in Suriname is much less pronounced than at the other sites, with monthly precipitation never falling below 100 mm. A recent analysis used remote sensing data to show that tropical forests in central and northeastern Amazonia, where mean annual precipitation exceeds 2000 mm, are able to sustain or enhance photosynthetic activity during the dry season (Guan et al. 2015), contrasting the notion of growth being limited by drought.

The second potential environmental stimulus for ring formation is insolation, which is known to have an important influence on tropical tree phenology (Borchert et al. 2005, 2015). For example, some species have been observed flushing their leaves twice a year at the Equator in response to two insolation peaks per year, and only once a year farther from the Equator where insolation has just one peak per year (Borchert et al. 2015; Calle et al. 2010). Daily insolation data are shown in Fig. 1 (green lines). The Suriname site is closest to the ‘insolation equator’ which, at ~3°N, is the latitude where insolation has the lowest year-round variation (Borchert et al. 2015). Across the other study sites, where *Cedrela* is known to exchange its leaves once per year and form annual rings, there is no clear relationship between insolation seasonality and *Cedrela* growth rhythm. Of these seven sites, some have two peaks of insolation per year (Ecuador, Venezuela and Manaus), and some just one peak of insolation per year (Bolivia, Aripuanã, Nova Iguaçu and Campeche; Fig. 1). Therefore, we believe that solar insolation is not the primary driver of the distinct biannual ring formation of *Cedrela* in Suriname. Furthermore, periods of leaf-fall do not consistently coincide with increasing, decreasing, peak or minimum insolation, though leaf-flush occurs more commonly when insolation is increasing or nearing its annual maximum (Fig. 1).

Controls on growth periodicity can also be endogenous (Bräuning et al. 2008a). Ring formation may not be a plastic response to an external cue but could instead be driven by a biologically determined growth rhythm. In other words, different populations of *Cedrela* may be adapted to shed their leaves at a specific time each year, coinciding with the local seasonality in water deficit. This is supported by observations of Costa et al. (2013) who showed that *Cedrela* growing in southern Brazil exhibited regular cambial dormancy during the dry season, even in years when there was no water deficit, thus implying some conservatism in growth behaviour. Furthermore, providence trials (where seeds sourced from different origins are grown under the same conditions) have shown that *Cedrela* from drier sites show more pronounced leaf-fall behaviour than *Cedrela* from wetter sites, indicating that variation in phenology is at least partly controlled by phylogeny (Newton et al. 1999). As *Cedrela odorata* is also known to have one of the highest levels of population differentiation of any tree species yet to be tested, with moist- and dry-adapted lineages (Cavers et al. 2003; Muellner et al. 2009), it seems feasible that regional differences in ring periodicity might be associated with phylogenetic differences.

**Fig. 4:**
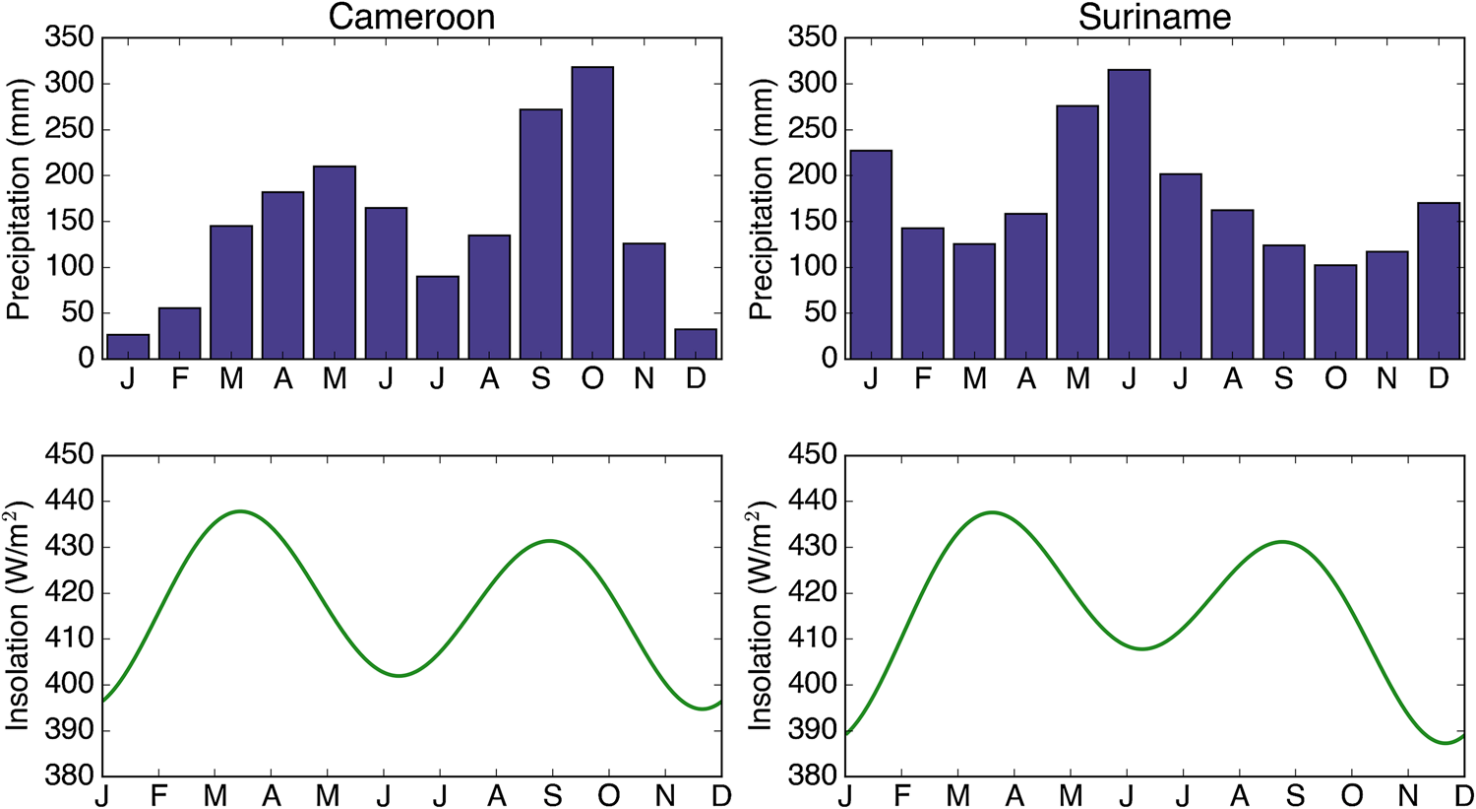
Annual precipitation (*upper panels*) and insolation (*lower panels*) in Cameroon and Suriname. Precipitation data are from CRU TS3.24 0.5° × 0.5° and insolation data were downloaded from http://data.giss.nasa.gov/ar5/srlocat.html

Finally, a comparison with *Cedrela* growing in plantations in Cameroon at a similar latitude (3.5°N) to the Suriname *Cedrela* (4.90°N) can provide further clues as to what controls tree growth in this species. The *Cedrela* trees from Cameroon are known to form annual rings (Détienne and Mariaux 1977), and as the annual course of insolation in Cameroon is almost identical to that in Suriname (Fig. 4) where rings are biannual, it is unlikely that insolation is the primary driver of *Cedrela* ring formation. Therefore, the differences in growth rhythm between Cameroon and Suriname must either be due to differences in climate or due to some internal (i.e. genetically controlled) growth rhythm of the plantation trees in Cameroon. As in Suriname, rainfall has a bimodal distribution in Cameroon (Fig. 4) but the dry periods in Cameroon are more extreme and trees only stop growing during the long dry season from December to February (Détienne and Mariaux 1977). As the Cameroon trees were most likely introduced from central America (most commercial *Cedrela* trees are), and as trees in Central America stop growing during the same period (December to February, e.g. Mexico, Fig. 1a), the distinct annual growth rhythm of the Cameroon trees may thus also be a genetic relict from the original population of these plantation trees. Indeed, differences in tree-ring periodicity between introduced and native tree species have been observed before in tropical Africa, and attributed to incomplete adaptation of the introduced species to the local climate (David et al. 2014). In conclusion, from our assessment of the available evidence, insolation is unlikely to be a driver of growth periodicity in *Cedrela*, but climate seasonality and/or genetics are likely to be important.

## Summary and outlook

Radiocarbon dating has been used to confirm that *Cedrela*, a tree widely used in tropical tree-ring studies, forms annual rings in Bolivia, Ecuador and Venezuela but two rings per year in Suriname. This result shows that annual tree-ring formation in a species at one site cannot automatically be extrapolated elsewhere. The rhythm of tree-ring formation at new locations needs to be established if tree rings are to be used for dating, especially in tropical sites with low climatic seasonality. With incomplete phenological data, it is difficult to draw definite conclusions about what controls *Cedrela* growth rhythms, though it seems that rainfall seasonality, not solar insolation, is the environmental cue triggering tree-ring formation, with a probable genetic influence. Phenological and growth rhythm observations of *Cedrela* in sites with relatively aseasonal climates like in Suriname, possibly combined with relocation experiments, would help us to better understand the spatial differences in growth dynamics of this scientifically and commercially important species.

### Author contribution statement

Sample preparation and ring analysis were conducted by JCAB with assistance from RJWB. GMS performed the radiocarbon analysis. JCAB wrote the paper and also prepared all of the figures. GMS wrote the methods section on radiocarbon analysis and all authors provided critical feedback on the manuscript.
